# Structural validity of the Norwegian version of the Strengths and Difficulties Questionnaire in children aged 3–6 years

**DOI:** 10.3389/fpsyg.2022.1024918

**Published:** 2022-12-14

**Authors:** Katrine Nyvoll Aadland, Arne Lervåg, Yngvar Ommundsen, Eivind Aadland

**Affiliations:** ^1^Department of Sport, Food and Natural Sciences, Faculty of Education, Arts and Sports, Western Norway University of Applied Sciences, Sogndal, Norway; ^2^Department of Pedagogy, Religion and Social Studies, Faculty of Education, Arts and Sports, Western Norway University of Applied Sciences, Sogndal, Norway; ^3^Department of Sport and Social Sciences, Norwegian School of Sport Sciences, Oslo, Norway

**Keywords:** mental health, preschool children, confirmatory factor analysis, psychometrics, SDQ

## Abstract

**Introduction:**

This study examined the structural validity of the teacher-report Strength and Difficulties Questionnaire (SDQ) in Norwegian preschoolers aged 3–6 years. We tested the original five-factor structure, the five-factor structure with two broader second-order factors, and a three-factor structure, all suggested in the literature. Since the positively worded items in SDQ have been shown to introduce noise, we also examined all three structures with a positive construal method factor for these items.

**Methods:**

Preschool teachers from 43 preschools completed the SDQ questionnaire for 1,142 children [48% girls, mean age 4.3 (SD 0.9) years]. Confirmatory factor analyses were used to estimate and compare the six models. Measurement invariance was tested across sex (multi-group approach) and age (multiple-indicator multiple-cause approach).

**Results:**

The original five-factor structure of SDQ was supported, where the model fit improved when including a method factor for positively worded items. Both models showed scalar invariance across sex and age. The second-order and the three-factor structures were not supported.

**Conclusion:**

We recommend using the original five-factor structure when using SDQ for both clinical and research purposes in young children and adding a method factor when using structural equation modeling.

## Introduction

The Strengths and Difficulties Questionnaire (SDQ) is a widely used instrument for measuring mental health in children and adolescents and is available in more than 80 languages.^1^ The questionnaire is developed for use in clinical and educational settings, as well as for screening and research (Goodman, 1997), and has parent-, teacher-, and self-report versions. SDQ was developed for children aged 3–16 years with an emphasis on strengths as well as difficulties to increase acceptability for use in general, healthy populations (Goodman, 1997). It assesses children’s behaviors, emotions, and relationships with 25 items equally divided between the five dimensions: conduct problems, emotional symptoms, hyperactivity, peer problems, and prosocial behavior (Goodman, 1997).

Validation studies generally support the original five-factor structure of SDQ across different languages and age groups (Smedje et al., 1999; Goodman, 2001; Van Roy et al., 2008; Stone et al., 2010; Mieloo et al., 2012; Niclasen et al., 2012; Ezpeleta et al., 2013; Croft et al., 2015; Kersten et al., 2016; McAloney-Kocaman and McPherson, 2017). A systematic review of 27 studies found strong evidence for the five-factor structure also in young children (Kersten et al., 2016). Seventeen of the included studies used confirmatory factor analysis (CFA) and were of fair quality. Nine of these studies had preschool teachers as informants of which eight studies reported acceptable to good model fit for the five-factor structure. The original studies that solely included young children (aged 3–7) used the SDQ versions in English (Hill and Hughes, 2007; Downs et al., 2012), German (Downs et al., 2012), Spanish (Hill and Hughes, 2007; Downs et al., 2012), Dutch (Mieloo et al., 2012), and Danish (Niclasen et al., 2012). More recent studies have also supported the five-factor structure of SDQ in Scottish (McAloney-Kocaman and McPherson, 2017) and Swedish (Dahlberg et al., 2019) samples.

Norwegian versions of the SDQ have been validated in different age groups (Rønning et al., 2004; Van Roy et al., 2008; Sanne et al., 2009; Bøe et al., 2016), but the structural validity of the Norwegian teacher-report form for preschool children is not known. Sanne et al. (2009) found an acceptable goodness of fit for Goodman’s five-factor model for both the Norwegian parent- and teacher-report versions of SDQ in children aged 7–9 years. Furthermore, the findings by Rønning et al. (2004), Van Roy et al. (2008), and Bøe et al. (2016) generally support a five-factor structure of the Norwegian self-report form for adolescents, but all studies suggest that modifications should be considered to improve its structural validity. More research is therefore needed to validate the structure of the Norwegian SDQ, particularly for young children.

Several alternative structures of the SDQ have been suggested. A highly cited study by Goodman et al. (2010) suggested using fewer, broader domains in the general population with low rates of disorders by grouping the items into one externalizing and one internalizing factor in addition to the prosocial behavior factor (Goodman et al., 2010). Ezpeleta et al. (2013) found support for both the original five-factor structure and a second-order model with internalizing and externalizing as second-order factors in 3-year-old children (using the SDQ version for 3- to 4-year-olds). However, others have found a better fit for the original five-factor structure than a three-factor structure (internalizing, externalizing, and prosocial behavior as factors) in preschoolers (Croft et al., 2015; McAloney-Kocaman and McPherson, 2017). Some studies have also suggested a six-factor structure that includes a method factor for the positively worded items (i.e., a “positive construal factor”), as these items tend to cluster together, in addition to the original five factors (Van Roy et al., 2008; McAloney-Kocaman and McPherson, 2017). Van Roy et al. (2008) identified a positive construal factor in their study of adolescents (10–19 years) but found that the factor had a modest role compared with the original factors. In a sample of 4-year-olds, McAloney-Kocaman and McPherson (2017) compared a three-factor model (with internalizing, externalizing, and prosocial behavior as factors), a five-factor model (with the original five factors), and a six-factor model (with a method factor in addition to the original five factors) for the standard parent-report SDQ in children and youth (3–16 years) and found that the six-factor solution provided the best model fit, although the original five-factor structure also was supported.

Some studies have tested differences in scores for the SDQ domains across sex and age (Downs et al., 2012; Mieloo et al., 2012; Niclasen et al., 2012). For example, Niclasen et al. (2012) found that boys scored higher on the hyperactivity, conduct problems, and peer problems scales and lower on the emotional symptoms and prosocial behavior scales compared to girls and that younger children scored higher on the hyperactivity and conduct problems scales compared to older children. However, few studies have examined the measurement invariance of the SDQ across sex and age, which is a prerequisite for such analyses. To the best of our knowledge, a study by Dahlberg et al. (2019), who found strong measurement invariance across sex, age, and parents’ education levels for parent- and teacher-report versions of SDQ in 3- to 5-year-olds, is the only previous study performing invariance testing of the SDQ structure in young children.

**FIGURE 1:**
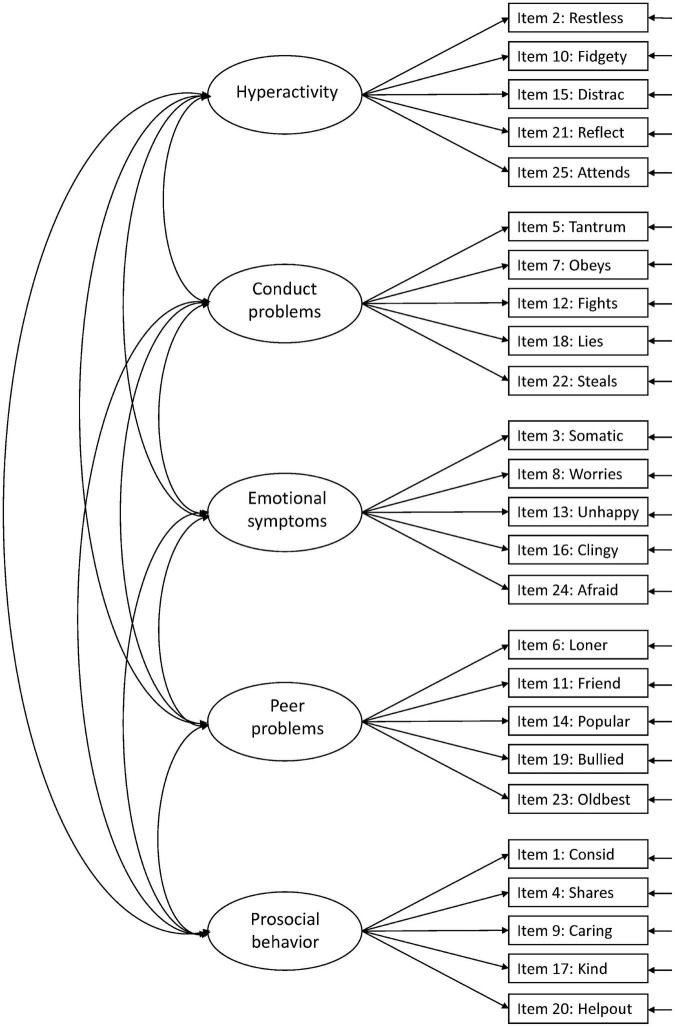
Original five-factor structure (Model 1).

**FIGURE 2:**
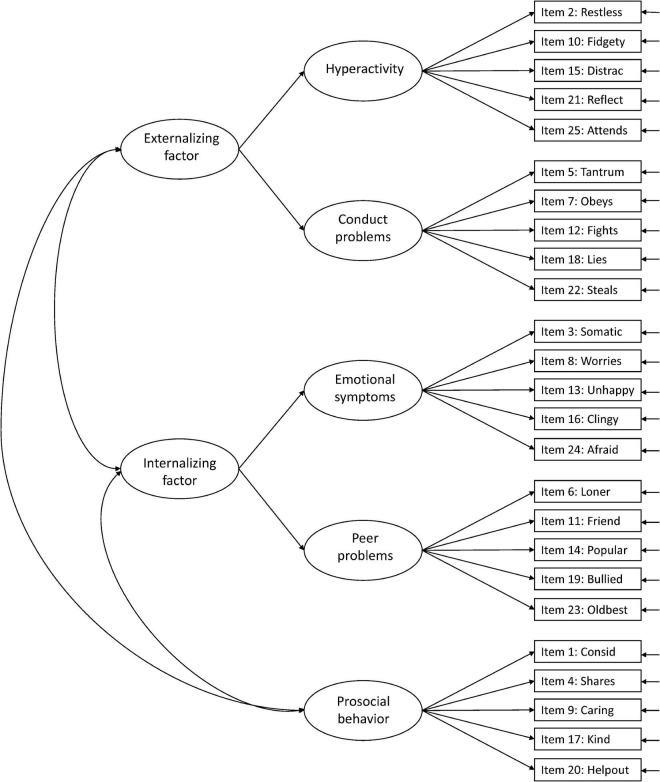
Second-order model with internalizing and externalizing as second-order factors from the original five-factor structure (Model 2).

**FIGURE 3:**
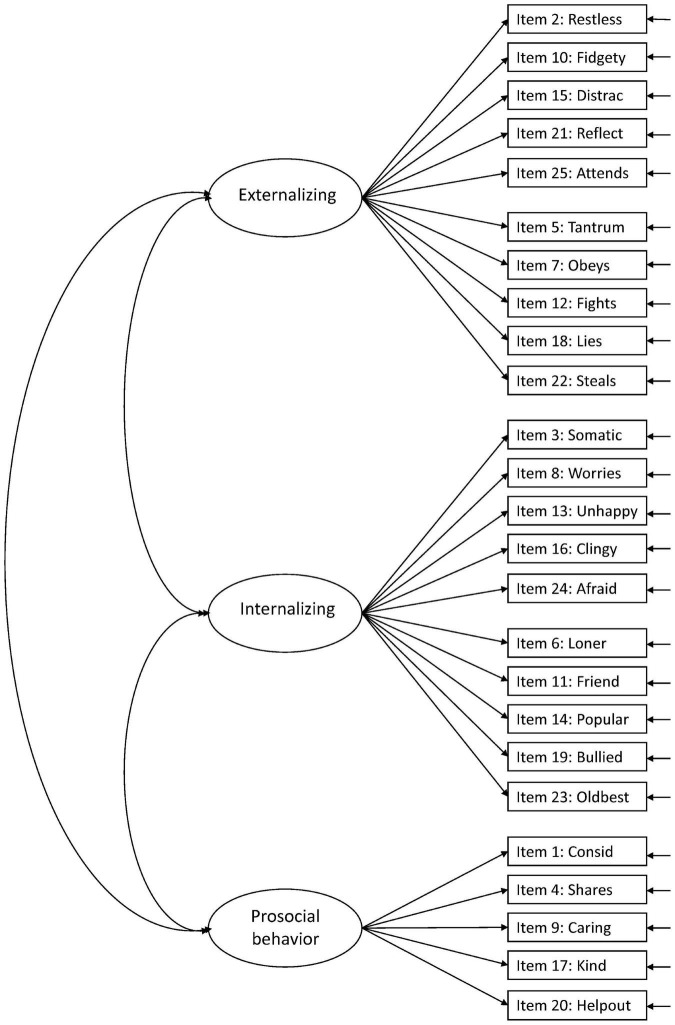
Three-factor model with internalizing, externalizing, and prosocial behavior as factors (Model 3).

**FIGURE 4:**
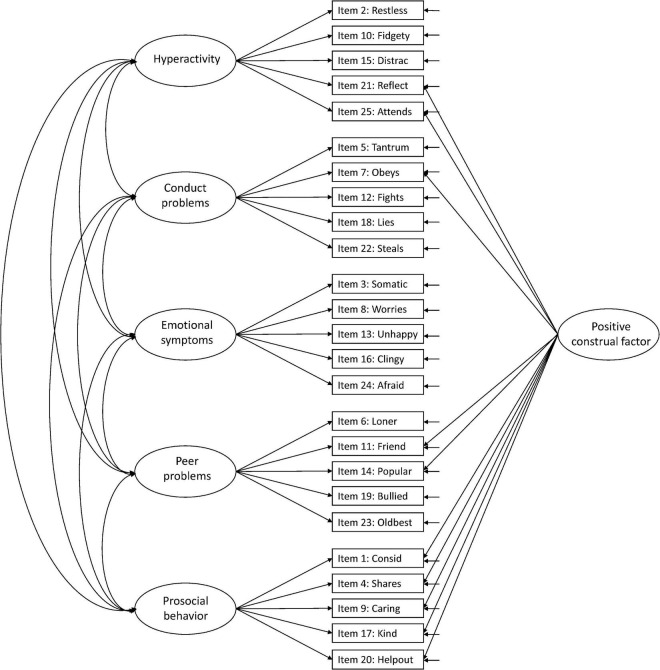
Original five-factor structure with a positive construal method factor for the positively worded items (Model 4).

**FIGURE 5:**
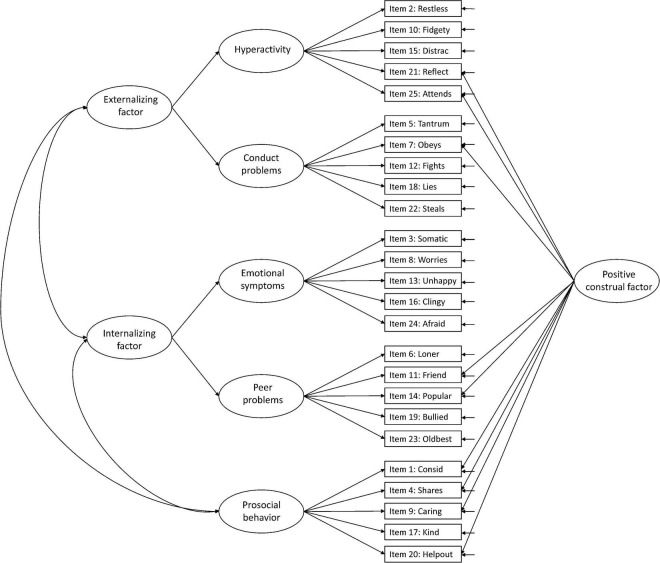
Second-order model with internalizing and externalizing as second-order factors from the original five-factor structure with a positive construal method factor for the positively worded items (Model 5).

**FIGURE 6:**
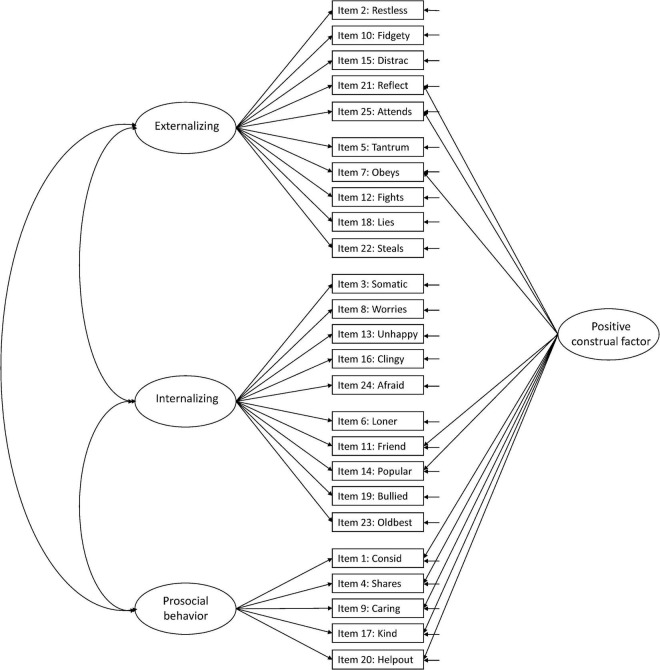
Three-factor model (internalizing, externalizing, and prosocial behavior) with a positive construal method factor for the positively worded items (Model 6).

Against this background, more research is needed to facilitate the interpretation of the SDQ across populations and purposes (McAloney-Kocaman and McPherson, 2017). In the present study, we aimed to examine the structural validity of the teacher-report SDQ in Norwegian preschoolers aged 3–6 years, including evaluation of measurement invariance over sex and age. We examined six hypothesized models in the prevailing literature: Model 1: the original five-factor structure (Goodman, 1997; Figure 1), Model 2: a second-order model with internalizing and externalizing as second-order factors in the original five-factor structure (Goodman et al., 2010; Figure 2), Model 3: a three-factor model with internalizing, externalizing, and prosocial behavior as factors (Figure 3; Goodman et al., 2010; McAloney-Kocaman and McPherson, 2017), Model 4: the original five-factor structure with a positive construal method factor for the positively worded items (Van Roy et al., 2008; McAloney-Kocaman and McPherson, 2017; Figure 4), Model 5: the second-order model with a method factor (Figure 5), and Model 6: the three-factor model (internalizing, externalizing, and prosocial behavior) with a method factor (Figure 6).

## Materials and methods

The present study used baseline data from the cluster randomized controlled trial *Active Learning Norwegian Preschool(er)s* (ACTNOW) (Aadland et al., 2020), collected during the autumn of 2019 and 2020. ACTNOW was conducted in the Sogn og Fjordane region in Western Norway and included 1,265 children (response rate 82%) aged 3–5 years from 46 preschools (response rate 82%) that fulfilled the inclusion criteria of having ≥ six 3- to 4-year-old children enrolled. Preschool teachers in three preschools refrained from completing the SDQ questionnaire; hence, 1,142 children (48% girls) attending 43 preschools were included in the present study.

### Strengths and Difficulties Questionnaire

The SDQ is a brief measure of psychosocial strengths and difficulties in 3- to 16-year-old children. The SDQ asks about 25 attributes (10 positives, 14 negatives, and one neutral attribute) across five domains of five items each: (1) emotional symptoms scale; (2) conduct problems scale; (3) hyperactivity scale; (4) peer problems scale; and (5) prosocial behavior scale. All but the last scale is also summed to provide a total difficulties score (range 0–40). SDQ uses a 3-point Likert scale to indicate the extent to which each attribute applies to a child [“not true” (score 0), “somewhat true” (score 1), or “certainly true” (score 2)] (Goodman, 1997, 2001). Positively worded items on scales 1–4 (item 7: *Obeys*, item 11: *Friend*, item 14: *Popular*, item 21: *Reflect*, and item 25: *Attends*) were scored reversely. The SDQ was scored according to the protocol (Goodman, 1997; see text footnote 1). We used the standard Norwegian (Nynorsk) version of the SDQ available from the SDQ website (see text footnote 1). Preschool teachers completed the questionnaire for each child enrolled in their department.

### Ethics statement

The procedures and methods used in ACTNOW conform to the ethical guidelines defined by the Word Medical Association’s Declaration of Helsinki and its subsequent revisions (World Medical Association, 2013). The study protocol was approved by the Norwegian Centre of Research Data (NSD) (reference number 248220) and the Institutional Ethics Committee. We obtained written consent from a parent or guardian of each child prior to the assessment.

### Statistical methods

Children’s age and difficulties scores are provided as mean and standard deviation (SD). These analyses were conducted using the SPSS software, version 27.0 (IBM SPSS Statistics for Windows, Armonk, NY: IBM Corp., USA).

Bivariate correlations between the five SDQ scales (sum scores), CFAs, and measurement invariance testing were conducted in Mplus, version 8 (Muthén and Muthén, Los Angeles, CA, USA). Due to the use of ordinal data and children being nested within preschools, the complex method with the robust weighted least squares mean and variance adjusted (WLSMV) estimator and delta parameterization was used in all CFAs and measurement invariance analyses. This also implies that missing data were handled by this estimator.

We examined the six hypothesized models illustrated in Figures 1–6. Due to the large sample size, multiple indices in addition to the chi-square test statistics (χ^2^) were used to assess model fit for the CFAs: A comparative fit index (CFI) and Tucker–Lewis index (TLI) ≥ 0.95, a root mean squared error of approximation (RMSEA) ≤ 0.06, and a standardized root mean squared residual (SRMR) ≤ 0.08 were considered good model fit (Hu and Bentler, 1999). In addition, we used modification indices to evaluate potential modifications to improve model fit. Internal consistency estimates are provided as coefficient omega (ω), estimated in Mplus.

As a secondary analysis, since items in the SDQ previously have been shown to cross-load (Goodman, 2001; Van Roy et al., 2008; Sanne et al., 2009) and studies have shown that the exploratory equation modeling (ESEM) approach is more flexible than the CFA approach (Asparouhov and Muthén, 2009; Xiao et al., 2019; Wei et al., 2022), we also used the ESEM syntax (with geomin rotations) to estimate the exploratory factor structure. Factor loadings ≥ 0.320 (Tabachnick and Fidell, 2001) were interpreted as substantive significant. We see these analyses as secondary, because they are solely data-driven and because our aim of the present study was to validate a questionnaire that has been developed from theory and is thoroughly validated and used in research and clinical practice for other age groups.

Models with good fit were tested for measurement invariance across age and between girls and boys. We used a multiple-indicator multiple-cause (MIMIC) approach to assess measurement invariance across age, as this approach allows for the use of age as a continuous variable. In the MIMIC models, the latent factors and the observed indicators were regressed on age in three steps as explained by Morin et al. (2016). In the first step (null model), age has no effect on the latent means, and the indicator intercepts as the direct paths for both the latent factors and the observed indicators are constrained to zero. In the second step (saturated model), we freely estimated the direct paths from age to all the observed indicators keeping the paths from age to the latent factors constrained to zero. In the third step (invariant model), we freely estimated the paths from age to the latent factors and age constrained the paths from age to the observed indicators to zero. We interpreted the results from these models such that if the saturated and invariant models had a better model fit than the null model, age was a predictor. If the saturated model had a better model fit than the invariant model, differential item functioning (DIF) existed as a function of age. If this was the case, partial invariance was estimated by including the direct effects of age on SDQ items showing large modification indices in addition to the direct effect on latent factors. Similar model fit indices for the saturated and invariant models supported measurement invariance for age.

We used a multi-group approach to measurement invariance testing across sex. To evaluate measurement invariance across sex, three nested models were compared by comparing increasingly restrictive models (i.e., b against a and c against b): (a) equivalence of the structure (configural invariance), (b) equivalence of factor loadings (weak invariance), and (c) equivalence of item intercepts (strong invariance).

We used a non-significant χ^2^ and the criteria proposed by Chen (2007) (*n* > 300) to indicate measurement invariance for both the MIMIC and nested models: ΔCFI < –0.010, supplemented by ΔRMSEA < 0.015 or ΔSRMR < 0.030 for loading/weak invariance, and ΔCFI < –0.010, supplemented by ΔRMSEA < 0.015 or ΔSRMR < 0.010 for intercept/strong invariance. A *p*-value ≤ 0.05 was used to indicate statistical significance in all analyses.

The outputs from all models are available from https://osf.io/rza5y/ (doi: 10.17605/OSF.IO/RZA5).

## Results

Children with data on at least one item on the SDQ were included in the analysis (*n* = 1142, 48% girls). We included 1130–1141 observations on each item (0.5% missing observations in total). The mean age of the children was 4.3 years (SD 0.9), and the mean difficulties score was 7.79 (SD 5.44) [6.50 (5.00) in girls and 8.98 (5.56) in boys]. Children’s scores are presented in Supplementary Table 1, and bivariate correlations between scales are presented in Table 1.

**TABLE 1:** Bivariate correlation matrix for all scales (sum scores) in Strength and Difficulties Questionnaire (SDQ).

	1	2	3	4	5
1. Hyperactivity scale	–				
2. Emotional symptoms scale	0.090	–			
3. Conduct problems scale	0.533	0.154	–		
4. Peer problems scale	0.356	0.246	0.322	–	
5. Prosocial behavior scale	–0.505	–0.136	–486	–0.385	–

All correlations are significant (*p* ≤ 0.01).

### Confirmatory factor analysis

**FIGURE 7:**
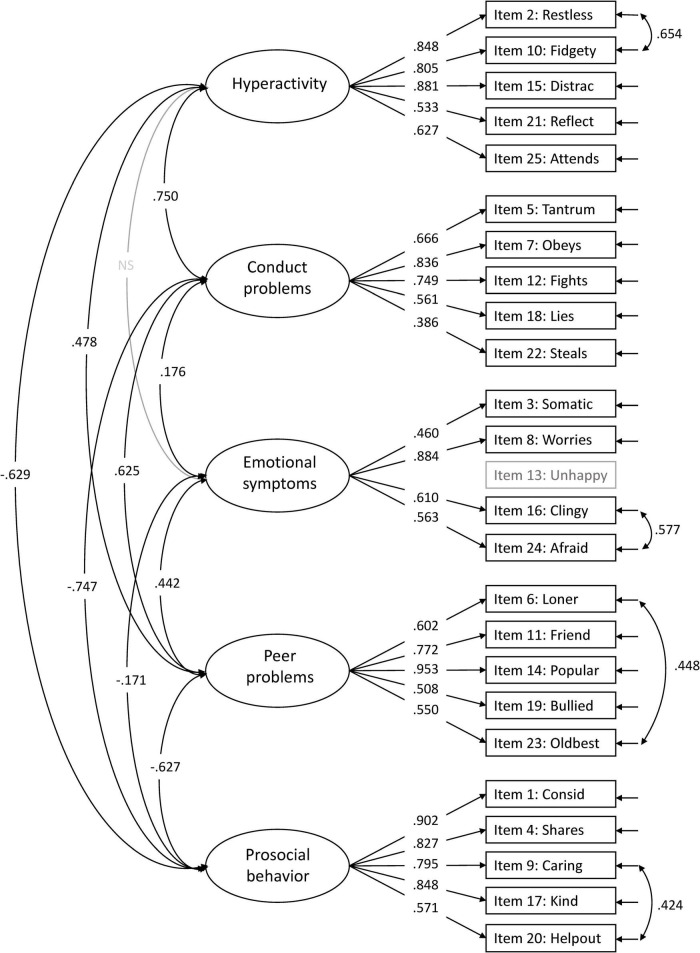
Accepted five-factor model (Model 1). All paths are significant unless marked non significant (NS). Item 13 is not included in the confirmatory factor analysis (CFA).

The original five-factor model (Model 1) suggested by Goodman (1997) showed a good model fit for CFI (0.954), RMSEA (0.035), and TLI (0.948) but not for SRMR (0.089) (Supplementary Figure 1). The internal consistency for the five factors was all above 0.80 (emotional symptoms; ω = 0.851, conduct problems; ω = 0.801, hyperactivity; ω = 0.903, peer problems; ω = 0.858, and prosocial behavior; ω = 0.903). Several modifications were suggested, with the highest modification index for item 13: *Unhappy*. This item was suggested to cross-load on all factors (modification indices range 55.33–71.82). These cross-loadings might indicate that item 13 is not well suited for the youngest children. The wording of the item is “Ofte lei seg, nedfor eller på gråten” (“Often unhappy, down-hearted or tearful”), where the last part, “på gråten” (“tearful”), possibly is problematic, since children at this age tend to cry for many reasons and in different situations. For this reason, we omitted item 13 in further analyses. Removing item 13 from the five-factor model resulted in a better model fit for all indices (CFI = 0.969, RMSEA = 0.030, TLI = 0.965, and SRMR = 0.080), all within the accepted criteria. The modification indices further suggested cross-loadings for some items and correlations between items. We allowed correlations for items with reasonable similarities within the same factor (i.e., item 2 with item 10, item 23 with item 6, item 9 with item 20, and item 24 with item 16). These minor modifications (correlations), except for the correlation between item 9 and item 20, were also seen in the five-factor models by Goodman et al. (2010). After taking these modifications into account, model fit indices for the original five-factor model (Model 1) with modifications were all within the criteria for good model fit (Table 2), with standardized factor loadings ≥ 0.386 (Figure 7).

**TABLE 2:** Model fit indices for the confirmatory factor analysis of the six tested models of the Strength and Difficulties Questionnaire (SDQ) structure (*n* = 1142).

Models	χ^2^ (*df*)	CFI	TLI	RMSEA (CI)	SRMR
Model 1: Five-factor model	639.063** (*265*)	0.954	0.948	0.035 (0.032–0.039)	0.089
Model 2: Second-order model	645.580** (*268*)	0.954	0.948	0.035 (0.032–0.039)	0.090
Model 3: Three-factor model	1052.090** (*272*)	0.904	0.894	0.050 (0.047–0.053)	0.118
Model 4: Five-factor model + method	548.371** (*255*)	0.964	0.958	0.032 (0.028–0.035)	0.080
Model 5: Second-order model + method	549.871** (*259*)	0.964	0.959	0.031 (0.028–0.035)	0.081
Model 6: Three-factor model + method	896.447** (*262*)	0.922	0.911	0.046 (0.043–0.049)	0.109
**Accepted models**					
Model 1: Five-factor model with modifications	425.140** (*238*)	0.977	0.973	0.026 (0.022–0.030)	0.073
Model 4: Five-factor model + method with modifications	348.252** (*228*)	0.985	0.982	0.021 (0.017–0.026)	0.063

**Significant *p* < 0.001. χ^2^, scaled chi-square fit statistics (under WLSMV); *df*, degrees of freedom; CFI, comparative fit index; TLI, Tucker–Lewis index; RMSEA, root mean square error of approximation; CI, 90% confidence interval; SRMR, standardized root mean square residual. Modifications in the accepted models are omitted item 13 and added correlations for items within the same factors (i.e., item 2 with item 10, item 23 with item 6; item 9 with item 20, item 24 with item 16).

**FIGURE 8:**
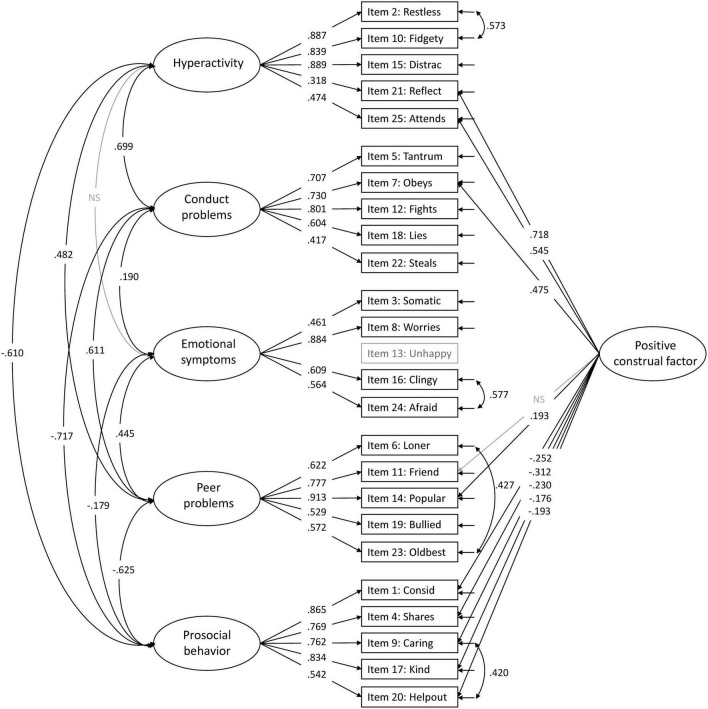
Accepted five-factor model with a positive construal method factor (Model 4). All paths are significant unless marked non significant (NS). Item 13 is not included in the confirmatory factor analysis (CFA).

Adding a positive construal factor for all positively worded items to the five-factor structure (Model 4) increased the model fit indices (Table 2). All positively worded items, except item 11: *Friend*, loaded significantly on the method factor, with factor loadings ranging from 0.176 to 0.718. Both items 21: *Reflect* and 25: *Attends* had higher factor loadings on the method factor than their original hyperactivity factor (Figure 8 for the model with modifications and Supplementary Figure 2 for the model without modifications).

The more parsimonious second-order model (Model 2), with internalizing and externalizing as second-order factors, did not have a different model fit than the original five-factor model (Table 2). However, we observed a Heywood case with a negative residual variance in the peer problems factor and a low correlation (0.40) between the two first-order factors for the internalizing factor, questioning the second-order structure. The same was found for the second-order model with a method factor (Model 5); it showed good model fit but a low correlation (0.43) between the first-order factors.

For the three-factor model (Model 3), model indices were poorer than for other models, and only the RMSEA was within the criteria for good model fit (Table 2). The variance for the internalizing factor was low (0.090), questioning its role in the model. The modification indices suggested several modifications. The highest modification index was suggested for the positively worded item 14: *Popular* on the prosocial behavior factor, which might be explained by common variance that will be accounted for in the three-factor model including the methods factor taking positively worded items into account. Hence, we did not add this cross-loading in the three-factor model. The correlation between items 16: *Clingy* and *24: Afraid* added in the other models was also included in the three-factor model. This modification gave a better model fit (Supplementary Table 2) but resulted in insignificant variance in the internalizing factor (0.076, *p* = 0.093). The internal consistency for the three factors was all above 0.87 (internalizing; ω = 0.871, externalizing; ω = 0.909, and prosocial behavior; ω = 0.903).

Adding a method factor to the three-factor model (Model 6) resulted in a better model fit than for Model 3; however, only the RMSEA was within the criteria for good model fit (Table 2). The model fit indices were better for the modified three-factor model with a method factor (correlation between item 16 and item 24), but still, only within the criteria of good model fit for the RMSEA (Supplementary Table 2). The variance in the internalizing factor was not significant. Factor loadings ranged between 0.301 and 0.951. However, the modification indices suggested a wide range of modifications.

Finally, we supplemented our accepted five-factor models (Models 1 and 4) from the CFA with an ESEM where all SDQ items were allowed to load on five factors. The model fit for the ESEM was good for all indices [CFI = 0.989, TLI = 0.981, RMSEA = 0.022 (0.017–0.027), and SRMR = 0.040] and similar to the model fit for the five-factor models. All items had the highest loading on their original factor, except for item 19: *Bullied*, which had higher loading on the conduct problems factor. In addition, we observed a cross-loading for item 2: *Restless* on the peer problems factor. All other cross-loadings were for the positively worded items on the prosocial behavior factor, which supports the inclusion of a method factor in SEM analysis of the SDQ (item 7: *Obeys*, also cross-loaded on the hyperactivity factor). All factor loadings for the ESEM are shown in Supplementary Table 3. Furthermore, model fit indices for all CFAs and ESEMs are shown in Supplementary Table 2.

### Measurement invariance

Since only the five-factor models (Models 1 and 4) were found to have good model fit, we only performed measurement invariance testing for these models. Both models showed scalar invariance both across sex (Table 3) and age (Table 4), showing that the structure of the SDQ did not differ between girls and boys and across the age range of 3–6 years. Age explained less than 2.3% of the variance in each of the original five factors (in Models 1 and 4) and 8.4% of the method factor [e.g., in the five-factor model with method factor (Model 4), the explained variances of age were 0.6, 0, 0.5, 1.7, 0.3, and 8.4% of the emotional symptoms, conduct problems, hyperactivity, peer problems, and prosocial behavior factors, and the method factor, respectively].

**TABLE 3:** Measurement invariance testing for the accepted models across sex.

Model comparison	Δχ^2^ (Δ*df)*	ΔCFI	ΔRMSEA	ΔSRMR
**Model 1: Five-factor model**				
Scalar against configural	59.899* (*38*)	0.001	–0.002	0.001
**Model 4: Five-factor model + method**				
Scalar against configural	79.184** (*46*)	0.000	–0.003	0.002

χ^2^, scaled chi-square fit statistic; *df*, degrees of freedom; CFI, comparative fit index; RMSEA, root mean square error of approximation; SRMR, standardized root mean square residual. *p < 0.05; **p < 0.001. Modifications in the accepted models are omitted item 13 and added correlations for items within the same factors (i.e., item 2 with item 10, item 23 with item 6; item 9 with item 20, item 24 with item 16).

**TABLE 4:** Multiple-indicator multiple-cause (MIMIC) models for age in accepted models.

Model comparison	χ^2^ (*df)*	CFI	RMSEA (CI)	SRMR	Δχ^2^ (Δ*df)*	ΔCFI	ΔRMSEA	ΔSRMR
**Model 1: Five-factor model**								
Age MIMIC null model	508.676** (*262*)	0.969	0.029 (0.025–0.032)	0.099				
Age MIMIC saturated	420.928** (*238*)	0.977	0.026 (0.022–0.030)	0.067	137.397** (24)	–0.008	0.003	0.032
Age MIMIC invariant	472.997** (*257*)	0.973	0.027 (0.023–0.031)	0.087	111.291** (19)	–0.004	0.001	0.020
**Model 4: Five-factor model + method**								
Age MIMIC null model	443.032** (*252*)	0.976	0.026 (0.022–0.030)	0.093				
Age MIMIC saturated	347.535** (*228*)	0.985	0.021 (0.017–0.026)	0.058	137.397** (*24*)	–0.009	0.004	0.035
Age MIMIC invariant	388.546** (*246*)	0.982	0.023 (0.018–0.027)	0.075	80.814** (*18*)	–0.003	0.002	0.017

Models 1 and 4 have the following modifications: omitted item 13 and added correlations for items within the same factors (i.e., item 2 with item 10, item 23 with item 6; item 9 with item 20, item 24 with item 16). CFI, comparative fit index; RMSEA, root mean square error of approximation; SRMR, standardized root mean square residual. **p < 0.001.

## Discussion

The present study aimed to examine the structural validity of teacher-reported SDQ in Norwegian preschoolers aged 3–6 years, including evaluation of measurement invariance over sex and age. By testing six hypothesized structural models in the prevailing literature, we found an acceptable model fit for the original five-factor structure (Goodman, 1997), and a superior fit for the original five-factor structure with a positive construal method factor for the positively worded items (Van Roy et al., 2008; McAloney-Kocaman and McPherson, 2017). Although the model fit for the second-order model was good, the low correlation between the first-order factors emotional symptoms and peer problems of the internalizing factor questions the convergent validity of this model. The model fit for the three-factor structure and the three-factor structure with a method factor was not supported. In addition, there was no significant variation among the children in the internalizing factor, questioning the validity of the three-factor model in the current sample. The five-factor structure—with and without a methods factor—had scalar invariance across sex and age (3–6 years).

Our findings support previous studies and confirm the validity of the original five-factor structure of the SDQ in preschool-aged children (Downs et al., 2012; Mieloo et al., 2012; Croft et al., 2015; Kersten et al., 2016; McAloney-Kocaman and McPherson, 2017; Dahlberg et al., 2019). Similar to previous studies, some modifications within the five-factor structure were indicated in the present study. As item 13: *Unhappy* cross-loaded on all factors, we chose to omit this item in our analysis because these cross-loadings might be explained by the fact that children of young age simply react with crying for a wide range of reasons, not only when being unhappy. Goodman (2001) also observed a cross-loading for item 13 but only to the conduct problems factor. However, their sample included older children (5–15 years). In addition to omitting item 13, we added minor modifications to the model, that is, four correlations between items of similar meaning/wording within the same factors. Three of these four correlations were the same minor modifications as in the five-factor model by Goodman et al. (2010). Generally, the small cross-loadings observed (and ignored) in the present and previous studies are expected as the children are young, and the domains may be less distinctive than in older children, making differentiation across sub-domains through preschool teacher reports more challenging. Van Roy et al. (2008) suggested that especially emotional and behavioral difficulties are less distinct constructs in young children than in older children.

Research has suggested using broader subscales for SDQ in low-risk, epidemiological samples (Goodman et al., 2010). Goodman et al. (2010) found support for a second-order model where the second-order factor of internalizing is indicated by the first-order factors emotional symptoms and peer problems, and externalizing by conduct problems and hyperactivity, for different SDQ forms and raters in children aged 5–15 years. In their study, the correlations between the first-order factors within the internalizing and externalizing factors were between 0.66–0.71 and 0.71–0.81, respectively. Our second-order model, including preschool children as rated by preschool teachers, showed very low convergent validity for the internalizing factor, where the correlation between the emotional symptoms and peer problems was 0.344. Goodman et al. (2010) further examined whether the five first-order factors could be replaced by the three factors internalizing, externalizing, and prosocial behavior but found a poor fit. Neither the present study nor McAloney-Kocaman and McPherson (2017) found support for a three-factor structure in preschool samples. The present study observed a lack of significant variation among the children in the internalizing factor. Together, evidence on the second-order model and the three-factor model suggests that these broader subscales should not be used in preschool children.

The internal consistency for the factors within the five-factor model was high in the present study with all coefficient Omegas above 0.80 (range 0.80–0.90). These findings are similar to those by Ezpeleta et al. (2013) reporting Omegas 0.91 for the total score and from 0.75 to 0.93 for the five domains of SDQ (version 3–4 years) in their sample of 3-year-old preschoolers. Other studies have mainly provided internal consistency coefficients such as Cronbach’s alpha, which are lower than Omega. In the systematic review by Kersten et al. (2016), Cronbach’s alpha for the teacher-report form of SDQ from 26 studies was on average 0.82 for the total score and ranged between 0.49 and 0.69 for the factors.

Although the present study supported the original five-factor structure, the inclusion of a method factor for the positively worded items provided a superior fit to the data. This finding is consistent with the finding by McAloney-Kocaman and McPherson (2017) using parent-reported SDQ in Scottish children, which to our knowledge is the only previous study examining this structural model in a sample of preschoolers. In both studies, the inclusion of a method factor resulted in lower factor loadings on the original factors for all positively worded items (in our study, not for item 11: *Friend*), and for some of these items also a lower factor loading on the original factor than on the method factor. In the present study, both items 21: *Reflect* and 25: *Attends* had higher factor loadings on the method factor than the original hyperactivity factor, whereas the Scottish study observed similar or higher factor loadings on the method factor than their original factors for all items except items 21 and 25 from the hyperactivity factor and item 7: *Obeys* from the conduct problems factor. These two studies’ findings indicate that the items with low loadings on the original factors after adding the method factor are less relevant as they do not provide substantive information for their factors. The significant factor loadings on the method factor, which for some items were high (ranges in the present study 0.17–0.54 and the Scottish study 0.32–0.51), might indicate that the positively worded items reflect method variance that needs to be accounted for in a separate positive construal method factor. Several previous studies have highlighted the noise associated with the positively worded items, but they all appear to agree that this noise is tolerable to gain acceptance for the use of the questionnaire in general healthy populations (Van Roy et al., 2008; McAloney-Kocaman and McPherson, 2017). In other words, although some of the positively worded items are less relevant for their factors, they should better be included to gain acceptability from the respondents.

The five-factor structure—with and without a method factor—was equal across sex and age, meaning that these groups’ latent means can be compared. This finding is consistent with the Swedish study by Dahlberg et al. (2019) examining the five-factor structure in preschool-aged children. To the best of our knowledge, no previous studies have examined the measurement invariance for the five-factor structure with a method factor.

The present study has several strengths and limitations. First, we included a large study sample, providing good evidence for the factor structure (including measurement invariance across sex and age) of the Norwegian teacher-report form of SDQ in young children. However, the sample was not representative of Norwegian preschoolers as children from a limited geographical area in the western part of Norway were included. Second, the strength of our analysis was the adjustment for the preschool level, as the same teacher completed the SDQ for all children attending their department in the preschool. A limitation of our study is that we included only teacher reports, while multi-informant approaches for both research and clinical purposes have been recommended (Stone et al., 2010). However, previous studies have shown that teacher-reported SDQ has higher levels of internal consistency than parent-reported SDQ (Kersten et al., 2016), and teachers in preschools have better opportunities than parents to judge how children relate to others (Stone et al., 2010).

## Conclusion and implications

The present study found support for the original five-factor structure of the SDQ (Goodman, 1997), but not a three-factor structure, in a sample of Norwegian preschoolers. However, we found a superior fit for a model with the five-factor structure including an additional positive construal method factor for the positively worded items. The inclusion of a method factor weakened the factor loadings for the positively worded items on their original factors. Despite the positively worded items that may provide measurement noise, it has been suggested that their inclusion may be important for acceptability for the SDQ by teachers completing the form. Thus, the present study points to the importance of including such a method factor to account for this noise. Good internal consistency (ω) for all factors (≥ 0.80) and measurement invariance across both sex and age provide evidence for the use of the five factors to compare latent means for sex and age groups. Hence, we recommend using the original five factors when using SDQ for both clinical and research purposes in young children and adding a method factor when using structural equation modeling. We further recommend excluding item 13: *Unhappy* when using SDQ in young children.

## Data availability statement

The raw data supporting the conclusion of this article will be made available by the authors, on reasonable request.

## Ethics statement

The study protocol was approved by the Norwegian Centre of Research Data (NSD) (reference number: 248220) and the Institutional Ethics Committee. Written informed consent to participate in this study was provided by the participants’ legal guardian/next of kin.

## Author contributions

KA performed the data collection, analyzed the data, and wrote the manuscript draft. EA obtained funding for the study, contributed to data analysis, and drafted the manuscript. AL contributed to data analysis and drafted the manuscript. YO drafted the manuscript. All authors conceived the idea for the manuscript, read, commented on, and approved the final manuscript.

## Footnotes

1
http://www.sdqinfo.org

## Supplementary material

Supplementary Figure 1 Confirmatory factor analysis of the five-factor model (no modifications).

Supplementary Figure 2 Confirmatory factor analysis of the five-factor model with a method factor (no modifications).

Supplementary Table 1 Proportions of scoring on each category for all items of Strength and Difficulties Questionnaire (SDQ).

Supplementary Table 2 Model fit indices for all tested models.

Supplementary Table 3 Exploratory equation modeling (ESEM) of Strength and Difficulties Questionnaire (SDQ).
