# Corrigendum: Structural validity of the Norwegian version of the Strengths and Difficulties Questionnaire in children aged 3–6 years

**DOI:** 10.3389/fpsyg.2023.1178961

**Published:** 2023-05-15

**Authors:** Katrine Nyvoll Aadland, Arne Lervåg, Yngvar Ommundsen, Eivind Aadland

**Affiliations:** ^1^Department of Sport, Food and Natural Sciences, Faculty of Education, Arts and Sports, Western Norway University of Applied Sciences, Sogndal, Norway; ^2^Department of Pedagogy, Religion and Social Studies, Faculty of Education, Arts and Sports, Western Norway University of Applied Sciences, Sogndal, Norway; ^3^Department of Sport and Social Sciences, Norwegian School of Sport Sciences, Oslo, Norway

**Keywords:** mental health, preschool children, confirmatory factor analysis, psychometrics, SDQ

In the published article, there was an error. In the published manuscript, re-coding of the positively worded items 7, 11, 14, 21, and 25 in the SDQ was erroneous. The response “Not true” was incorrectly coded as “Somewhat true,” and vice versa. The authors have corrected the error in the datafile and reanalyzed the data. In the correction, all estimates provided in text, tables, and figures have been corrected. Some minor corresponding corrections have been done in the discussion. The conclusion is not changed.

A correction has been made to **Results**, Paragraph 1. The corrected paragraph is shown below.

“Children with data on at least one item on the SDQ were included in the analysis (*n* = 1,142, 48% girls). We included 1,130–1,141 observations on each item (0.5% missing observations in total). The mean age of the children were 4.3 years (SD 0.9), and the mean difficulties score was 6.42 (SD 5.04) [5.22 (4.35) in girls and 7.54 (5.36) in boys]. Children's scores are presented in Supplementary Table 1, and bivariate correlations between scales are presented in Table 1.”

**Supplementary Table 1:** Proportions and counts (in brackets) of scoring on each category for all items of SDQ.

**SDQ subscales and items**	**Categories of scoring**		
	**Not true**	**Somewhat true**	**Certainly true**
**Hyperactivity**			
SDQ_2: Restless	63.3 (719)	25.9 (294)	10.8 (123)
SDQ_10: Fidgety	61.3 (693)	27.0 (305)	11.7 (132)
SDQ_15: Distrac	44.2 (502)	39.4 (448)	16.4 (186)
SDQ_21: Reflect^*^	12.9 (146)	55.0 (625)	32.1 (365)
SDQ_25: Attends^*^	11.9 (135)	47.1 (536)	41.0 (467)
**Conduct problems**			
SDQ_5: Tantrum	83.1 (946)	13.4 (152)	3.5 (40)
SDQ_7: Obeys^*^	5.7 (65)	37.1 (423)	57.2 (651)
SDQ_12: Fights	85.3 (966)	12.4 (141)	2.3 (26)
SDQ_18: Lies	87.4 (989)	10.7 (121)	1.9 (22)
SDQ_22: Steals	96.7 (1,093)	2.5 (28)	0.8 (9)
**Emotional symptoms**			
SDQ_3: Somatic	94.9 (1,083)	4.6 (53)	0.4 (5)
SDQ_8: Worries	87.6 (998)	11.1 (126)	1.3 (15)
SDQ_13: Unhappy	84.7 (960)	13.5 (153)	1.9 (21)
SDQ_16: Clingy	59.7 (697)	30.9 (390)	9.4 (106)
SDQ_24: Afraid	78.1 (889)	19.1 (218)	2.8 (32)
**Peer problems**			
SDQ_6: Loner	84.3 (958)	13.3 (151)	2.4 (27)
SDQ_11: Friend^*^	4.4 (50)	12.7 (144)	82.9 (941)
SDQ_14: Popular^*^	1.0 (11)	20.0 (227)	79.0 (897)
SDQ_19: Bullied	91.6 (1,040)	7.9 (90)	0.4 (5)
SDQ_23: Oldbest	71.7 (812)	23.1 (262)	5.2 (59)
**Prosocial behavior**			
SDQ_1: Consid^*^	2.5 (28)	41.9 (477)	55.6 (633)
SDQ_4: Shares^*^	5.3 (60)	55.5 (633)	39.3 (448)
SDQ_9: Caring^*^	4.4 (50)	43.2 (492)	52.4 (596)
SDQ_17: Kind^*^	0.5 (6)	19.9 (226)	79.6 (905)
SDQ_20: Helpout^*^	16.0 (182)	50.0 (567)	34.0 (385)

* Positively worded items (not reversed scorings).

**Table 1:** Bivariate correlation matrix for all scales (sum scores) in SDQ.

	**1**	**2**	**3**	**4**	**5**
1. Hyperactivity scale	-				
2. Emotional symptoms scale	0.088	-			
3. Conduct problems scale	0.531	0.168	-		
4. Peer problems scale	0.346	0.241	0.323	-	
5. Prosocial behavior scale	−0.504	−0.132	−0.490	−0.385	-

All correlations are significant (p ≤ 0.01).

Corrections have been made to **Results**, “*Confirmatory factor analysis*,” Paragraphs 1–6. The corrected paragraphs are shown below.

“The original five-factor model (model 1) suggested by Goodman (1997) showed good model fit for CFI (0.958) RMSEA (0.037) and TLI (0.953), but not for SRMR (0.086; Supplementary Figure 1). The internal consistency for the five factors were all above 0.80 (Emotional symptoms; ω = 0.853, Conduct problems; ω = 0.811, Hyperactivity; ω = 0.942, Peer problems; ω = 0.843, and Prosocial behavior; ω = 0.904). Several modifications were suggested, with the highest modification index for item 13: *Unhappy*. This item was suggested to cross-load on all factors (modification indices range 66.03–83.53). These cross-loadings might indicate that item 13 is not well-suited for the youngest children. The wording of the item is “Ofte lei seg, nedfor eller på gråten” (“Often unhappy, down-hearted or tearful”), where the last part, “på gråten” (“tearful”), possibly is problematic, since children at this age tend to cry for many reasons and in different situations. For this reason, we omitted item 13 in further analyses. Removing item 13 from the five-factor model resulted in better model fit for all indices (CFI = 0.972, RMSEA = 0.032, TLI = 0.968, and SRMR = 0.075), all within the accepted criteria. The modification indices further suggested cross-loadings for some items and correlations between items. We allowed correlations for items with reasonable similarities within the same factor (i.e., item 2 with item 10, item 23 with item 6, item 9 with item 20, and item 24 with item 16). These minor modifications (correlations), except for the correlation between item 9 and item 20, were also seen in the five-factor models by Goodman et al. (2010). After taking these modifications into account, model fit indices for the original five-factor model (model 1) with modifications were all within the criteria for good model fit (Table 2), with standardized factor loadings ≥ 0.404 (Figure 7).

**Supplementary Figure 1:**
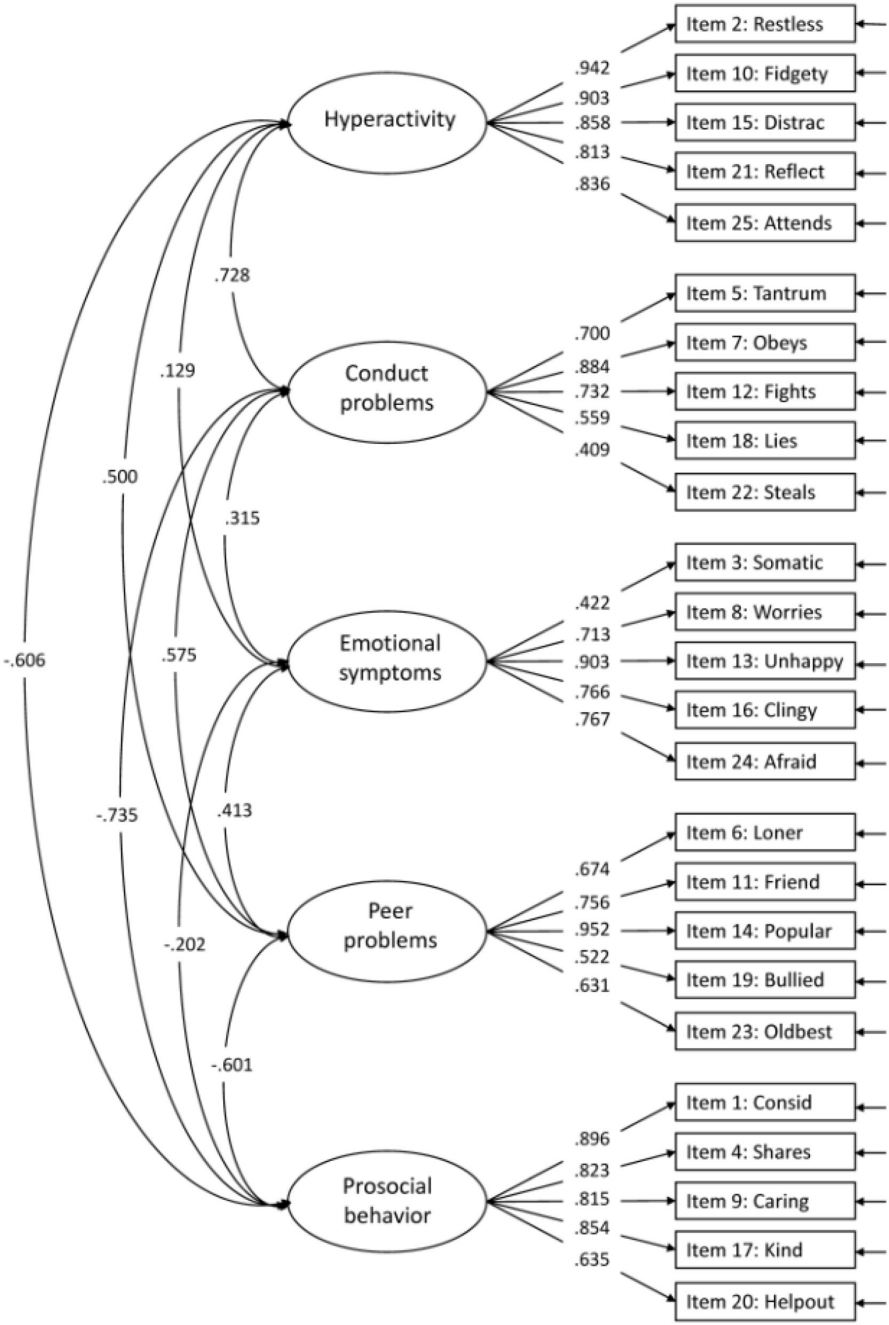
Confirmatory Factor Analysis of the five-factor model (no modifications).

**Table 2:** Model fit indices for the confirmatory factor analysis of the six tested models of the SDQ structure (*n* = 1,142).

**Models**	**χ^2^*(df)***	**CFI**	**TLI**	**RMSEA (CI)**	**SRMR**
Model 1: Five-factor model	682.914^**^ (*265*)	0.958	0.953	0.037 (0.034–0.041)	0.086
Model 2: Second-order model	688.057^**^ (*268*)	0.958	0.953	0.037 (0.034–0.040)	0.087
Model 3: Three-factor model	1,171.162^**^ (*272*)	0.910	0.901	0.054 (0.051–0.057)	0.115
Model 4: Five-factor model + method	600.259^**^ (*255*)	0.966	0.960	0.034 (0.031–0.038)	0.079
Model 5: Second-order model + method	596.257^**^ (*259*)	0.966	0.961	0.034 (0.030–0.037)	0.081
Model 6: Three-factor model + method	981.477^**^ (*262*)	0.928	0.918	0.049 (0.046–0.052)	0.108
**Accepted models**					
Model 1: Five-factor model with modifications	431.320^**^ (*238*)	0.981	0.978	0.027 (0.023–0.031)	0.068
Model 4: Five-factor model + method with modifications	387.757^**^ (*228*)	0.984	0.981	0.025 (0.020–0.029)	0.064

** Significant p < 0.001.

χ^2^, scaled chi-square fit statistics (under WLSMV); df, degrees of freedom; CFI, Comparative Fit Index; TLI, Tucker-Lewis Index; RMSEA, Root Mean Square Error of Approximation; CI, 90% Confidence Interval; SRMR, Standardized Root Mean Square Residual.

Modifications in the accepted models are omitted item 13 and added correlations for items within the same factors (i.e., item 2 with item 10, item 23 with item 6; item 9 with item 20, item 24 with item 16).

**Figure 7:**
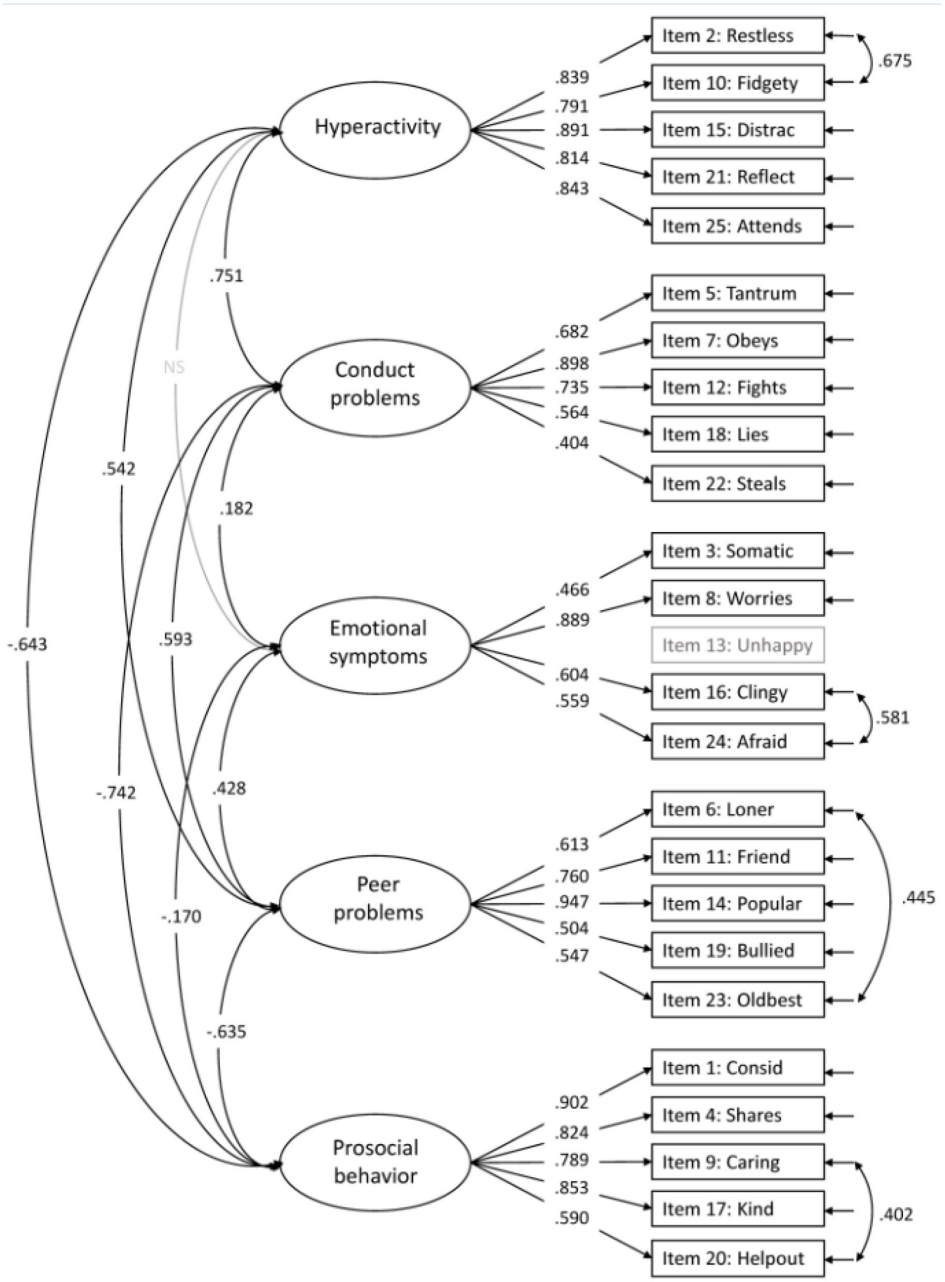
The accepted five-factor model (model 1). All paths are significant unless marked NS. Item 13 is not included in the CFA.

Adding a positive construal factor for all positively worded items to the five-factor structure (Model 4) increased the model fit indices (Table 2). All positive items loaded significantly on the method factor, with factor loadings ranging from 0.154–0.558 (Figure 8 for model with modifications and Supplementary Figure 2 for model without modifications).

**Figure 8:**
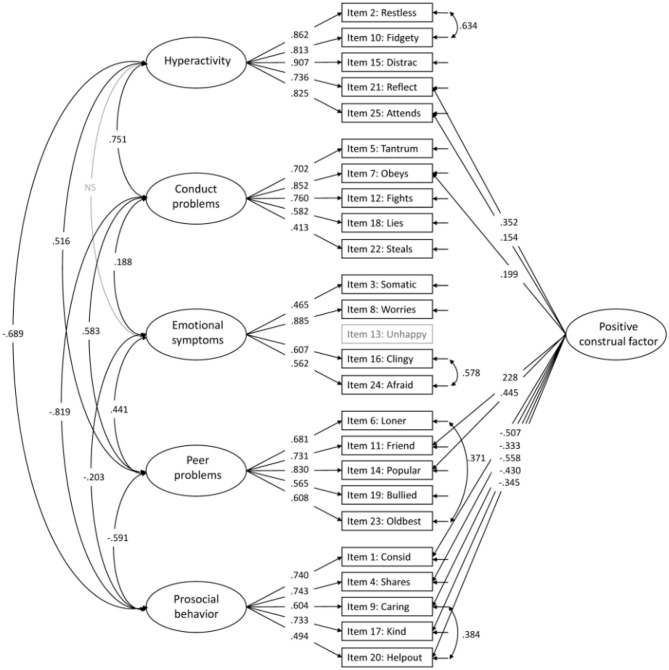
The accepted five-factor model with a positive construal method factor (model 4). All paths are significant unless marked NS. Item 13 is not included in the CFA.

**Supplementary Figure 2:**
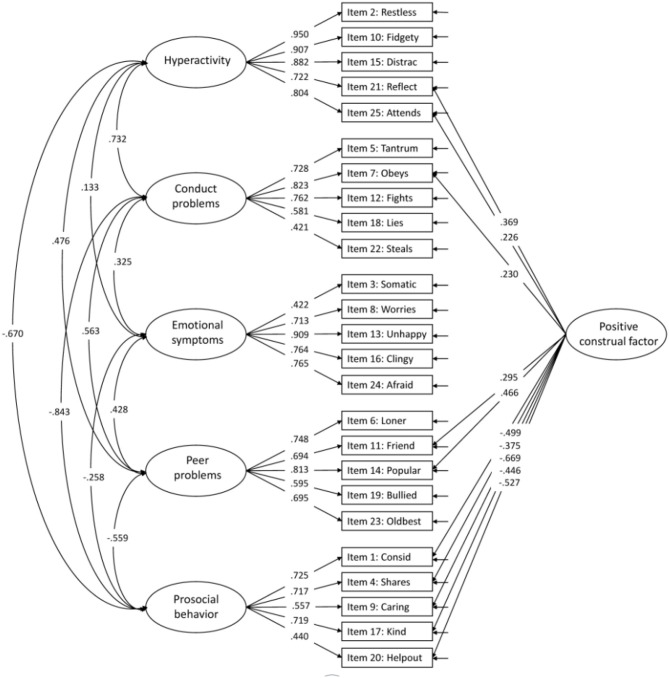
Confirmatory Factor Analysis of the five-factor model with a method factor (no modifications).

The more parsimonious second-order model (Model 2), with internalizing and externalizing as second-order factors, did not have a different model fit than the original five-factor model (Table 2). However, we observed a Heywood case with a negative residual variance in the Peer problems factor and a low correlation (0.39) between the two first-order factors for the Internalizing factor, questioning the second-order structure. The same was found for the second-order model with a method factor (Model 5); it showed good model fit but a low correlation (0.43) between the first-order factors.

For the three-factor model (Model 3), model indices were poorer than for other models, and only the RMSEA was within the criteria for good model fit (Table 2). The variance for the Internalizing factor was low (0.091), questioning its role in the model. The modification indices suggested several modifications. The highest modification index was suggested for the positively worded item 14: *Popular* on the prosocial behavior factor, which might be explained by common variance that will be accounted for in the three-factor model including the methods factor taking positively worded items into account. Hence, we did not add this cross-loading in the three-factor model. The correlation between items 16: *Clingy* and *24: Afraid* added in the other models was also included in the three-factor model. This modification gave better model fit (Supplementary Table 2) but resulted in insignificant variance in the Internalizing factor (0.075, *p* = 0.159). The internal consistency for the three factors were all above 0.86 (Internalizing; ω = 0.863, Externalizing; ω = 0.929, and Prosocial behavior; ω = 0.903).

**Supplementary Table 2:** Model fit indices for the confirmatory and exploratory factor analysis of the six tested models of the SDQ structure (*n* = 1,142) without and with modifications.

**Model**	**χ^2^*(df)***	**CFI**	**TLI**	**RMSEA (CI)**	**SRMR**
Model 1: Five-factor model^#^	682.914^**^ (*265*)	0.958	0.953	0.037 (0.034–0.041)	0.086
Model 1: Five-factor model without item 13	525.443^**^ (*242*)	0.972	0.968	0.032 (0.028–0.036)	0.075
Model 1: Five-factor model without item 13 and with correlations^#^	431.320^**^ (*238*)	0.981	0.978	0.027 (0.023–0.031)	0.068
Model 2: Second-order model^#^	688.057^**^ (*268*)	0.958	0.953	0.037 (0.034–0.040)	0.087
Model 2: Second-order model with Peer@0	677.083^**^ (*269*)	0.959	0.955	0.036 (0.033–0.040)	0.087
Model 2: Second-order model without item 13 and with Peer@0	521.826^**^ (*246*)	0.973	0.969	0.031 (0.028–0.035)	0.076
Model 3: Three-factor model^#^	1,174.162^**^ (*272*)	0.910	0.901	0.054 (0.051–0.057)	0.115
Model 3: Three-factor model with correlation for items 16 and 24	921.742^**^ (*271*)	0.935	0.928	0.046 (0.043–0.049)	0.103
Model 3: Three-factor model without item 13	1,075.756 (*249*)	0.918	0.909	0.054 (0.051–0.057)	0.111
Model 4: Five-factor model + method^#^	600.259^**^ (*255*)	0.966	0.960	0.034 (0.031–0.038)	0.079
Model 4: Five-factor model + method without item 13	443.807^**^ (*232*)	0.979	0.975	0.028 (0.024–0.032)	0.069
Model 4: Five-factor model + method without item 13 and with correlations^#^	387.757^**^ (*228*)	0.984	0.981	0.025 (0.020–0.029)	0.064
Model 5: Second-order model + method^#^	596.257^**^ (*259*)	0.966	0.961	0.034 (0.030–0.037)	0.081
Model 5: Second-order model + method without item 13	442.163^**^ (*236*)	0.980	0.976	0.028 (0.024–0.032)	0.070
Model 5: Second-order model + method without item 13 and with correlations	391.76^**^ (*232*)	0.984	0.981	0.025 (0.020–0.029)	0.065
Model 6: Three-factor model + method^#^	981.477^**^ (*262*)	0.928	0.918	0.049 (0.046–0.052)	0.108
Model 6: Three-factor model with correlation for item 16 and 24	780.803^**^ (*261*)	0.948	0.940	0.042 (0.038–0.045)	0.096
Model 6: Three-factor model + method without item 13	890.352^**^ (*239*)	0.935	0.925	0.049 (0.045–0.052)	0.106
EFA	274.192^**^ (*185*)	0.991	0.986	0.021 (0.015–0.026)	0.038
EFA without item 13	239.001^**^ (*166*)	0.993	0.988	0.020 (0.014–0.025)	0.036

** Significant p < 0.001.

# , also presented in manuscript; χ^2^, scaled chi-square fit statistics (under WLSMV); df, degrees of freedom; CFI, Comparative Fit Index; TLI, Tucker-Lewis Index; RMSEA, Root Mean Square Error of Approximation; CI, 90% Confidence Interval; SRMR, Standardized Root Mean Square Residual.

Adding a method factor to the three-factor model (Model 6) resulted in a better model fit than for Model 3; however, only the RMSEA was within the criteria for good model fit (Table 2). The model fit indices were better for the modified three-factor model with a method factor (correlation between item 16 and item 24), but still, only within the criteria of good model fit for the RMSEA (Supplementary Table 2). The variance in the internalizing factor was not significant. Factor loadings ranged between 0.285 and 0.941. However, the modification indices suggested a wide range of modifications.

Finally, we supplemented our accepted five-factor models (Models 1 and 4) from the CFA with an ESEM where all SDQ items were allowed to load on five factors. The model fit for the ESEM was good for all indices [CFI = 0.991, TLI = 0.986, RMSEA = 0.021 (0.015–0.026), and SRMR = 0.038] and similar to the model fit for the five-factors models. Cross-loadings were mainly observed for indicators within the Conduct problem and Prosocial behavior factors, which is also evident in the CFA, where the discriminant validity between these factors was low. All factor loadings for the ESEM are shown in Supplementary Table 3. Furthermore, model fit indices for all CFAs and ESEMs are shown in Supplementary Table 2.”

**Supplementary Table 3:** Exploratory Factor Analysis (EFA) of the SDQ.

	**Conduct problems scale**	**Hyperactivity scale**	**Emotional symptoms scale**	**Peer problems scale**	**Prosocial behavior scale**
SDQ_1: Consid	**−0.904** ^*^	0.025	0.046	−0.015	**0.458** ^*^
SDQ_2: Restless	0.002	**0.891** ^*^	0.003	0.113^*^	**0.356** ^*^
SDQ_3: Somatic	0.234	−0.203	**0.363** ^*^	0.005	0.010
SDQ_4: Shares	**−0.754** ^*^	−0.072	−0.012	−0.021	**0.361**
SDQ_5: Tantrum	**0.634** ^*^	0.020	0.136^*^	0.047	0.098
SDQ_6: Loner	−0.077	0.037	0.212	**0.816** ^*^	0.006
SDQ_7: Obeys	**0.645** ^*^	**0.302** ^*^	−0.044	−0.054	0.007
SDQ_8: Worries	0.024	−0.084	**0.649** ^*^	0.259^*^	0.001
SDQ_9: Caring	**−0.774** ^*^	−0.003	0.002	−0.008	**0.707** ^*^
SDQ_10: Fidgety	0.039	**0.882** ^*^	−0.005	0.008	**0.328** ^*^
SDQ_11: Friend	0.196	0.004	−0.069	**0.731** ^*^	−0.070
SDQ_12: Fights	**0.797** ^*^	0.023	0.025	−0.116	0.119
SDQ_13: Unhappy					
SDQ_14: Popular	**0.416** ^*^	0.016	−0.002	**0.542** ^*^	−0.092
SDQ_15: Distrac	−0.023	**0.891** ^*^	0.185^*^	0.031	0.008
SDQ_16: Clingy	0.004	0.004	**0.920** ^*^	−0.007	**−0.431**
SDQ_17: Kind	**−0.886** ^*^	0.038	−0.006	−0.005	**0.420** ^*^
SDQ_18: Lies	**0.486** ^*^	0.130	−0.016	0.044	0.135
SDQ_19: Bullied	**0.412** ^*^	−0.044	0.059	0.178	0.292^*^
SDQ_20: Helpout	**−0.482** ^*^	−0.163^*^	−0.006	0.025	**0.566** ^*^
SDQ_21: Reflect	**0.412** ^*^	**0.0498** ^*^	−0.070	−0.029	−0.006
SDQ_22: Steals	0.283^*^	0.121	0.120	−0.014	0.207^*^
SDQ_23: Oldbest	0.007	−0.034	0.278^*^	**0.700** ^*^	0.058
SDQ_24: Afraid	−0.015	0.003	**0.889** ^*^	−0.010	**−0.370** ^*^
SDQ_25: Attends	0.003	**0.812** ^*^	0.207^*^	−0.091	−0.112^*^

* p < 0.05. Green = items included in the original factor. Bold = factor loadings of substantive significance [0.320 and above (Tabachnick and Fidell, 2001)]. Item 13 omitted from the EFA.

A correction has been made to **Results**, “*Measurement invariance*.” The corrected paragraph is shown below.

“Since only the five-factor models (Models 1 and 4) were found to have good model fit, we only performed measurement invariance testing for these models. Both models showed scalar invariance both across sex (Table 3) and age (Table 4), showing that the structure of the SDQ did not differ between girls and boys and across the age range of 3–6 years. Age explained <2.0% of the variance in each of the original five factors (in model 1 and 4) and 9.9% of the method factor (e.g., in the five-factor model with method factor (model 4), the explained variances of age were 0.6, 0, 0.8, 0.7, 0.1, and 9.9% of the Emotional symptoms, Conduct problems, Hyperactivity, Peer problems, and Prosocial behavior factors and the method factor, respectively).”

**Table 3:** Measurement invariance testing for the accepted models across sex.

**Model comparison**	**Δχ^2^ (Δ*df)***	**ΔCFI**	**ΔRMSEA**	**ΔSRMR**
**Model 1: Five-factor model**				
Scalar against configural	51.176 (*38*)	0.002	−0.002	0.001
**Model 4: Five-factor model** + **method**				
Scalar against configural	59.952 (*46*)	0.002	−0.002	0.002

χ^2^, scaled chi-square fit statistic; df, degrees of freedom; CFI, Comparative Fit Index; RMSEA, Root Mean Square Error of Approximation; SRMR, Standardized Root Mean Square Residual.

Modifications in the accepted models are omitted item 13 and added correlations for items within the same factors (i.e., item 2 with item 10, item 23 with item 6; item 9 with item 20, item 24 with item 16).

**Table 4:** Multiple-indicators multiple-causes (MIMIC) models for age in accepted models.

**Model comparison**	**χ^2^ (*df)***	**CFI**	**RMSEA (CI)**	**SRMR**	**Δχ^2^ (Δ*df)***	**ΔCFI**	**ΔRMSEA**	**ΔSRMR**
**Model 1: Five-factor model**								
Age MIMIC null model	512.436^**^ (*262*)	0.974	0.029 (0.025–0.033)	0.094				
Age MIMIC saturated	428.461^**^(*238*)	0.981	0.026 (0.022–0.030)	0.063	125.730^**^ (*24*)	−0.007	0.003	0.031
Age MIMIC invariant	475.968^**^ (*257*)	0.978	0.027 (0.023–0.031)	0.081	97.843^**^ (*19*)	−0.003	0.001	0.017
**Model 4: Five-factor model** + **method**								
Age MIMIC null model	475.320^**^ (*252*)	0.977	0.028 (0.024–0.032)	0.092				
Age MIMIC saturated	387.116^**^ (*228*)	0.984	0.025 (0.020–0.029)	0.059	125.730^**^(*24*)	−0.007	0.003	0.033
Age MIMIC invariant	431.829^**^ (*246*)	0.981	0.026 (0.022–0.030)	0.075	92.435^**^ (*18*)	−0.003	0.001	0.016

Model 1 and 4 have the following modifications: omitted item 13 and added correlations for items within the same factors (i.e., item 2 with item 10, item 23 with item 6; item 9 with item 20, item 24 with item 16).

CFI, Comparative Fit Index; RMSEA, Root Mean Square Error of Approximation; SRMR, Standardized Root Mean Square Residual.

** p < 0.001.

A correction has also been made to **Discussion**, Paragraphs 3–5. The corrected paragraphs are shown below.

“Research has suggested using broader subscales for SDQ in low-risk, epidemiological samples (Goodman et al., 2010). Goodman et al. (2010) found support for a second-order model where the second-order factor of internalizing is indicated by the first-order factors emotional symptoms and peer problems, and externalizing by conduct problems and hyperactivity, for different SDQ forms and raters in children aged 5–15 years. In their study, the correlations between the first-order factors within the internalizing and externalizing factors were between 0.66–0.71 and 0.71–0.81, respectively. Our second-order model including preschool children as rated by preschool teachers, showed very low convergent validity for the Internalizing factor, where the correlation between the Emotional symptoms and Peer problems was 0.39. Goodman et al. (2010) further examined whether the five first-order factors could be replaced by the three factors internalizing, externalizing, and prosocial behavior but found a poor fit. Neither the present study nor McAloney-Kocaman and McPherson (2017) found support for a three-factor structure in preschool samples. The present study observed a lack of significant variation among the children in the internalizing factor. Together, evidence on the second-order model and the three-factor model suggests that these broader subscales should not be used in preschool children.

The internal consistency for the factors within the five-factor model were high in the present study with all coefficient Omegas above 0.80 (range 0.81–0.94). These findings are similar to those by Ezpeleta et al. (2013) reporting Omegas 0.91 for the total score and from 0.75 to 0.93 for the five domains of SDQ (version 3–4 years) in their sample of 3-year-old preschoolers. Other studies have mainly provided internal consistency coefficients such as Cronbach's alpha, which are lower than Omega. In the systematic review by Kersten et al. (2016), Cronbach's alpha for the teacher-report form of SDQ from 26 studies was on average 0.82 for the total score and ranged between 0.49 and 0.69 for the factors.

Although the present study supported the original five-factor structure, the inclusion of a method factor for the positively worded items provided a superior fit to the data. This finding is consistent with the finding by McAloney-Kocaman and McPherson (2017) using parent-reported SDQ in Scottish children, which to our knowledge is the only previous study examining this structural model in a sample of preschoolers. In both studies, inclusion of a method factor resulted in lower factor loadings on the original factors for all positive worded items. The significant factor loadings on the method factor, which for some items were high (ranges in the present study 0.15–0.56 and in the Scottish study 0.32–0.51) might indicate that the positive worded items reflect method variance that needs to be accounted for in a separate positive construal method factor. Several previous studies have highlighted the noise associated with the positively worded items, but they all appear to agree that this noise is tolerable to gain acceptance for the use of the questionnaire in general healthy populations (Van Roy et al., 2008; McAloney-Kocaman and McPherson, 2017). In other words, although some of the positively worded items are less relevant for their factors, they should better be included to gain acceptability from the respondents.”

In the published article, there were also errors in Tables 2–4, and Figures 7, 8, Supplementary Tables 1–3 and Supplementary Figures 1, 2, in relation to the issues outlined above. The corrected tables and figures are shown below.

The authors apologize for these errors and state that they do not change the scientific conclusions of the article in any way. The original article and Supplementary material have been updated.
