# Supplementary figures and images for: Structural validity of the Norwegian version of the Strengths and Difficulties Questionnaire in children aged 3–6 years
Source: Front Psychol. 2022 Dec 14;13:1024918. doi: 10.3389/fpsyg.2022.1024918 (PMC9795199; doi:10.3389/fpsyg.2022.1024918)

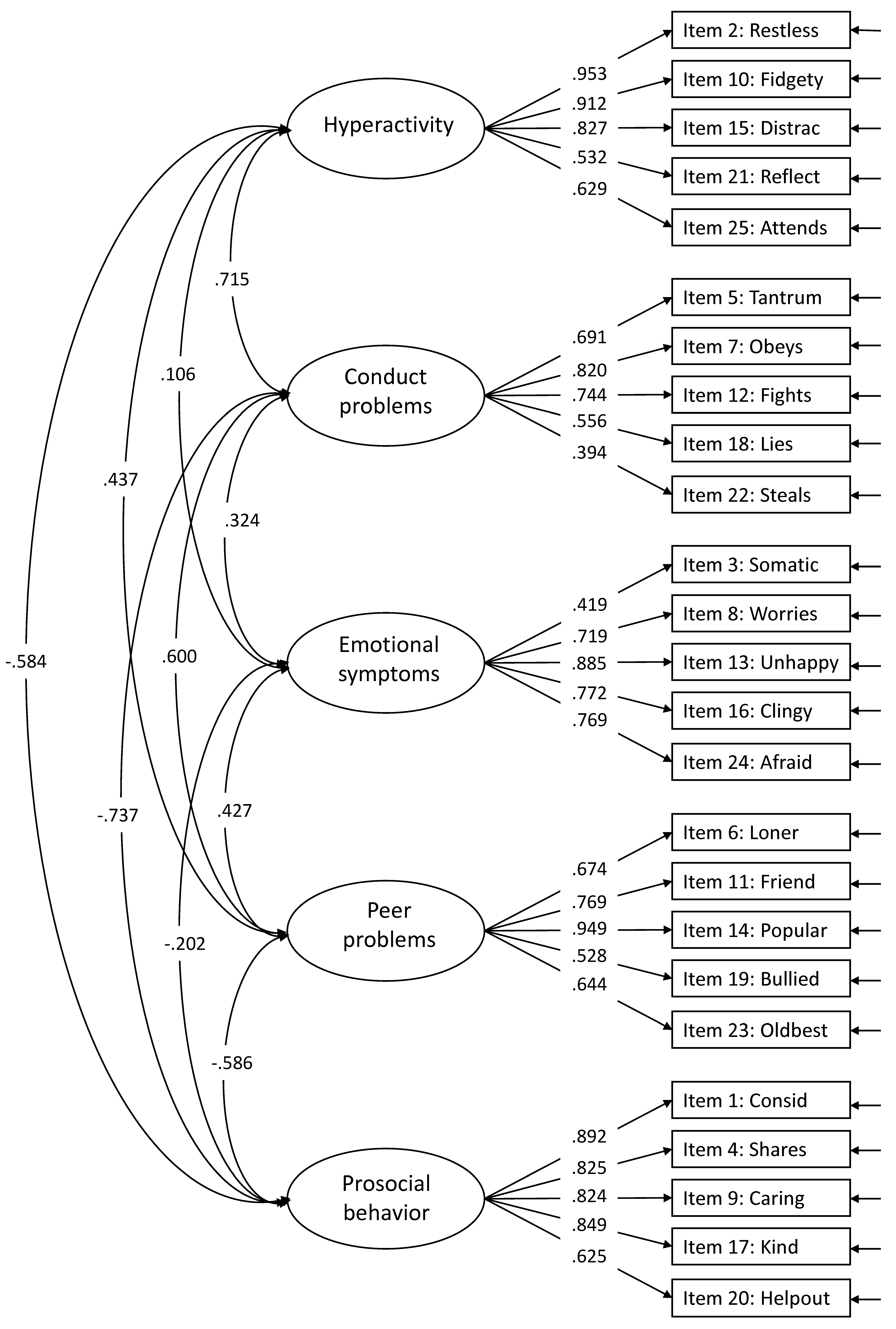

Supplement: Supplementary Figure 1 — Confirmatory factor analysis of the five-factor model (no modifications). [file Image_1.TIFF]

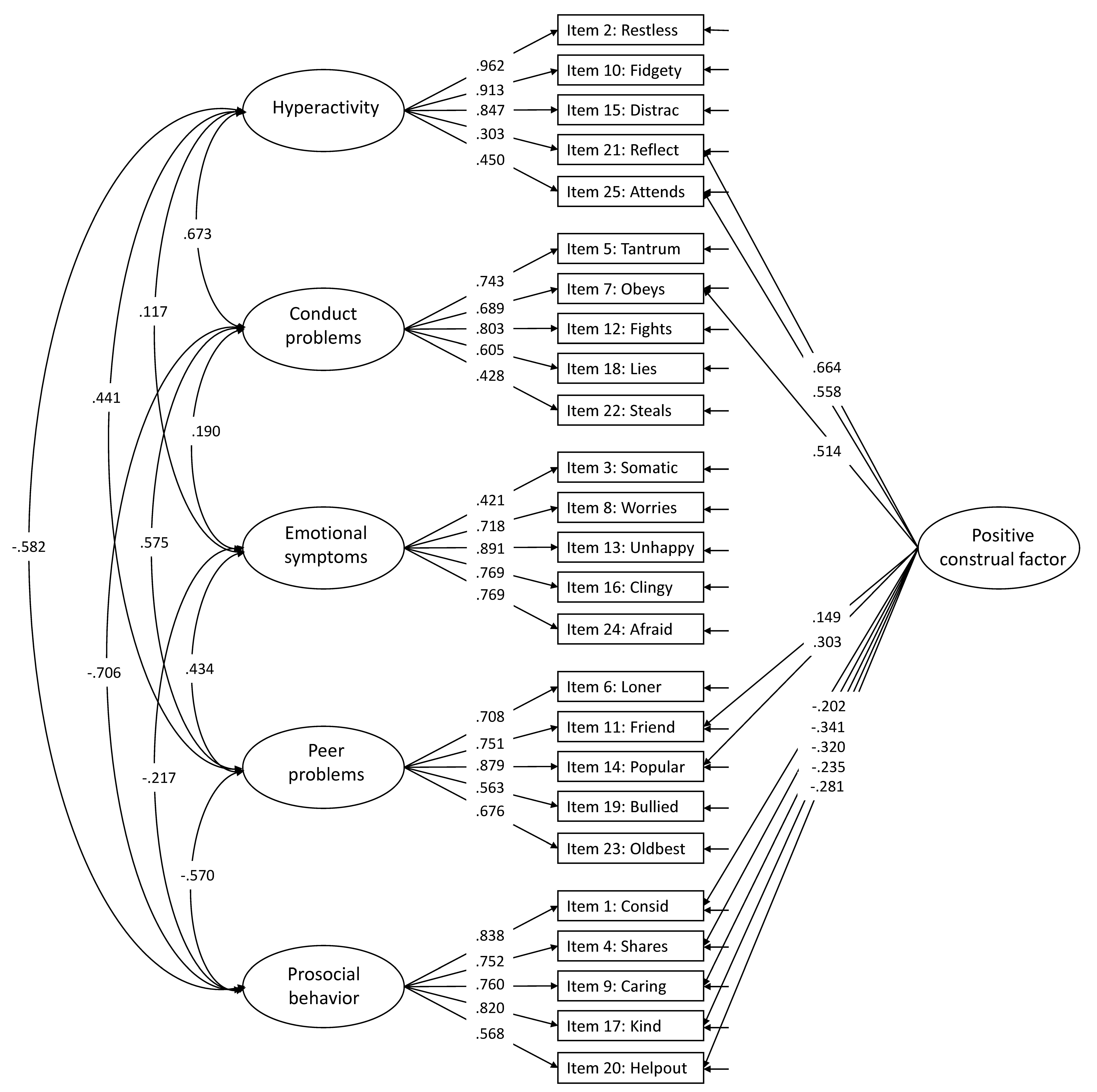

Supplement: Supplementary Figure 2 — Confirmatory factor analysis of the five-factor model with a method factor (no modifications). [file Image_2.TIFF]
