# Supplementary material for: Structural validity of the Norwegian version of the Strengths and Difficulties Questionnaire in children aged 3–6 years
Source: Front Psychol. 2022 Dec 14;13:1024918. doi: 10.3389/fpsyg.2022.1024918 (PMC9795199; doi:10.3389/fpsyg.2022.1024918)
Supplement: Supplementary Table 1 — Proportions of scoring on each category for all items of Strength and Difficulties Questionnaire (SDQ). [file Table_1.pdf]

Supplementary Material

Supplemental table 1. Proportions and counts (in brackets) of scoring on each category for all items of SDQ.

**SDQ subscales** and items	**Categories of scoring**		
	**Not True**	**Somewhat True**	**Certainly True**
**Hyperactivity**			
SDQ_2: Restless	63.3 (719)	25.9 (294)	10.8 (123)
SDQ_10: Fidgety	61.3 (693)	27.0 (305)	11.7 (132)
SDQ_15: Distrac	44.2 (502)	39.4 (448)	16.4 (186)
SDQ_21: Reflect*	55.0 (625)	12.9 (146)	32.1 (365)
SDQ_25: Attends*	47.1 (536)	11.9 (135)	41.0 (467)
**Conduct problems**			
SDQ_5: Tantrum	83.1 (946)	13.4 (152)	3.5 (40)
SDQ_7: Obeys*	37.1 (463)	5.7 (65)	57.2 (651)
SDQ_12: Fights	85.3 (966)	12.4 (141)	2.3 (26)
SDQ_18: Lies	87.4 (989)	10.7 (121)	1.9 (22)
SDQ_22: Steals	96.7 (1093)	2.5 (28)	.8 (9)
**Emotional symptoms**			
SDQ_3: Somatic	94.9 (1083)	4.6 (53)	.4 (5)
SDQ_8: Worries	87.6 (998)	11.1 (126)	1.3 (15)
SDQ_13: Unhappy	84.7 (960)	13.5 (153)	1.9 (21)
SDQ_16: Clingy	59.7 (697)	30.9 (390)	9.4 (106)
SDQ_24: Afraid	78.1 (889)	19.1 (218)	2.8 (32)
**Peer problems**			
SDQ_6: Loner	84.3 (958)	13.4 (151)	3.5 (27)
SDQ_11: Friend*	12.7 (144)	4.4 (50)	82.9 (941)
SDQ_14: Popular*	20.0 (227)	1.0 (11)	79.0 (897)
SDQ_19: Bullied	91.6 (1040)	7.9 (90)	.4 (5)
SDQ_23: Oldbest	71.7 (812)	23.1 (262)	5.2 (59)
**Prosocial behavior**			
SDQ_1: Consid*	2.5 (28)	41.9 (477)	55.6 (633)
SDQ_4: Shares*	5.3 (60)	55.5 (633)	39.3 (448)
SDQ_9: Caring*	4.4 (50)	43.2 (492)	52.4 (596)
SDQ_17: Kind*	.5 (6)	19.9 (226)	79.6 (905)
SDQ_20: Helpout*	16.0 (182)	50.0 (567)	34.0 (385)

Note. * Positively worded items (not reversed scorings).
