# Supplementary material for: Structural validity of the Norwegian version of the Strengths and Difficulties Questionnaire in children aged 3–6 years
Source: Front Psychol. 2022 Dec 14;13:1024918. doi: 10.3389/fpsyg.2022.1024918 (PMC9795199; doi:10.3389/fpsyg.2022.1024918)
Supplement: Supplementary Table 2 — Model fit indices for all tested models. [file Table_2.pdf]

Supplementary Material

Supplemental table 2. Model fit indices for the confirmatory and exploratory factor analysis of the six tested models of the SDQ structure (n=1142) without and with modifications.

Model	χ^2(*df*)	CFI	TLI	RMSEA (CI)	SRMR
**Model 1:** Five-factor model#	639.063** (*265*)	.954	.948	.035 (.032–.039)	.089
**Model 1:** Five-factor model without item 13	493.518** (*242*)	.969	.965	.030 (.026–.034)	.080
**Model 1:** Five-factor model without item 13 and with correlations#	425.140** (*238*)	.977	.973	.026 (.022–.030)	.073
**Model 2:** Second-order model#	645.580** (*268*)	.954	.948	.035 (.032–.039)	.090
**Model 2:** Second-order model with Peer@0	639.348** (*269*)	.955	.949	.035 (.031–.038)	.090
**Model 2:** Second-order model without item 13 and with Peer@0	492.816** (*246*)	.970	.966	.030 (.026–.033)	.081
**Model 3:** Three-factor model#	1052.090** (*272*)	.904	.894	.050 (.047–.053)	.118
**Model 3:** Three-factor model with correlation for items 16 and 24	837.724** (*271*)	.930	.923	.043 (.040–.046)	.106
**Model 3:** Three-factor model without item 13	947.518** (*249*)	.914	.905	.050 (.046–.053)	.115
**Model 4:** Five-factor model + method#	548.371** (*255*)	.964	.958	.032 (.028–.035)	.080
**Model 4:** Five-factor model + method without item 13	403.487** (*232*)	.979	.975	.025 (.021–.030)	.069
**Model 4:** Five-factor model + method without item 13 and with correlations#	348.252** (*228*)	.985	.982	.021 (.017–.026)	.063
**Model 5:** Second-order model + method#	549.871** (*259*)	.964	.959	.031 (.028–.035)	.081
**Model 5:** Second-order model + method without item 13	403.217** (*236*)	.979	.976	.025 (.021–.029	.070
**Model 5:** Second-order model + method without item 13 and with correlations	354.700** (*232*)	.985	.982	.022 (.017–.026)	.064
**Model 6:** Three-factor model + method#	896.447** (*262*)	.922	.911	.046 (.043–.049)	.109
**Model 6:** Three-factor model with correlation for item 16 and 24	716.697** (*261*)	.944	.936	.039 (.036–.043)	.096
**Model 6:** Three-factor model + method without item 13	804.933** (*239*)	.931	.920	.046 (.042–.049)	.107
**ESEM**	294.298** (*185*)	.987	.978	.023 (.018–.028)	.044
**ESEM without item 13**	259.111*' (*166*)	.989	.981	.022 (.017–.027)	.040

Note: ** significant p <.001; # = also presented in manuscript; χ^2 = scaled chi-square fit statistics (under WLSMV); *df* = degrees of freedom; CFI = Comparative Fit Index; TLI = Tucker-Lewis Index; RMSEA = Root Mean Square Error of Approximation; CI = 90 % Confidence Interval; SRMR = Standardized Root Mean Square Residual
