# Supplementary material for: Structural validity of the Norwegian version of the Strengths and Difficulties Questionnaire in children aged 3–6 years
Source: Front Psychol. 2022 Dec 14;13:1024918. doi: 10.3389/fpsyg.2022.1024918 (PMC9795199; doi:10.3389/fpsyg.2022.1024918)
Supplement: Supplementary Table 3 — Exploratory equation modeling (ESEM) of Strength and Difficulties Questionnaire (SDQ). [file Table_3.pdf]

Supplementary Material

Supplemental Table 3: Exploratory Structural Equation Modeling (ESEM) of the SDQ.

	Prosocial behavior scale	Hyperactivity scale	Emotional symptoms scale	Peer problems scale	Conduct problems scale
**SDQ_1: Consid**	**.810***	.024	.027	-.010	-.240*
**SDQ_2: Restless**	.024	**.797***	-.009	**.469***	.130*
**SDQ_3: Somatic**	.004	-.148	**.338***	-.018	.302*
**SDQ_4: Shares**	**.692***	-.072	-.026	-.012	-.233*
**SDQ_5: Tantrum**	-.267*	.030	.131*	.085	**.539***
**SDQ_6: Loner**	-.016	.036	.161	**.835***	-.174*
**SDQ_7: Obeys**	**-.459***	**.347***	-.047	-.020	.308*
**SDQ_8: Worries**	.095	-.043	**.583***	.240*	.211*
**SDQ_9: Caring**	**.886***	.013	-.009	.001	.015
**SDQ_10: Fidgety**	-.005	**.798***	.003	.333*	.156*
**SDQ_11: Friend**	-.253	-.052	-.033	**.669***	.007
**SDQ_12: Fights**	**-.328***	.008	.007	-.051	**.754***
**SDQ_13: Unhappy**					
**SDQ_14: Popular**	**-.400***	.007	-.007	**.524***	.163*
**SDQ_15: Distrac**	-.146	**.725***	.136*	.311*	-.003
**SDQ_16: Clingy**	-.103	.026	**.873***	-.006	-.018
**SDQ_17: Kind**	**.753***	.048	-.018	-.004	-.290*
**SDQ_18: Lies**	-.199	.082	-.046	.014	**.527***
**SDQ_19: Bullied**	-.009	-.069	.010	.225	**.573***
**SDQ_20: Helpout**	**.665***	-.120*	-.012	-.018	.126*
**SDQ_21: Reflect**	**-.395***	**.373***	-.026	-.161*	.006
**SDQ_22: Steals**	-.004	.024	.072	.070	**.488***
**SDQ_23: Oldbest**	.009	.001	.232*	**.688***	-.012
**SDQ_24: Afraid**	-.048	.010	**.818***	-.001	.041
**SDQ_25: Attends**	**-.379***	**.516***	.151*	-.004	-.157*

Note * p <.05. Green = items included in the original factor. Bold = factor loadings of substantive significance (.320 and above [Tabachnick & Fidell, 2001]). Item 13 omitted from the EFA.
